# LaMnO_3_-Type Perovskite Nanofibers as Effective Catalysts for On-Cell CH_4_ Reforming via Solid Oxide Fuel Cells

**DOI:** 10.3390/molecules29153654

**Published:** 2024-08-01

**Authors:** Yangbo Jia, Tong Wei, Zhufeng Shao, Yunpeng Song, Xue Huang, Beila Huang, Chen Cao, Yufan Zhi

**Affiliations:** 1 School of Materials Science & Engineering, Zhejiang SCI-TECH University, Hangzhou 310018, China; 2021316101121@mails.zstu.edu.cn (Y.J.); 2022316101093@mails.zstu.edu.cn (C.C.); 2023316101096@mails.zstu.edu.cn (Y.Z.); 2 China Industrial Energy Conservation and Cleaner Production Association, Beijing 100034, China; shaozhufeng163@163.com; 3 Industry Development Center of Zhejiang Province, Hangzhou 310006, China; 15397109810@189.cn; 4 Zhejiang Institute of Industry and Information Technology, Hangzhou 310006, China; 15067154645@163.com

**Keywords:** on-cell CH_4_ reforming, anode of solid oxide fuel cell, LCMN nanofibers, morphology improvement in reforming layer, stability

## Abstract

CH_4_ has become the most attractive fuel for solid oxide fuel cells due to its wide availability, narrow explosion limit range, low price, and easy storage. Thus, we present the concept of on-cell reforming via SOFC power generation, in which CH_4_ and CO_2_ can be converted into H_2_ and the formed H_2_ is electrochemically oxidized on a Ni-BZCYYb anode. We modified the porosity and specific surface area of a perovskite reforming catalyst via an optimized electrostatic spinning method, and the prepared LCMN nanofibers, which displayed an ideal LaMnO_3_-type perovskite structure with a high specific surface area, were imposed on a conventional Ni-BZCYYb anode for on-cell CH_4_ reforming. Compared to LCMN nanoparticles used as on-cell reforming catalysts, the NF-SOFC showed lower ohmic and polarization resistances, indicating that the porous nanofibers could reduce the resistances of fuel gas transport and charge transport in the anode. Accordingly, the NF-SOFC displayed a maximum power density (MPD) of 781 mW cm^−2^ and a stable discharge voltage of around 0.62 V for 72 h without coking in the Ni-BZCYYb anode. The present LCMN NF materials and on-cell reforming system demonstrated stability and potential for highly efficient power generation with hydrocarbon fuels.

## 1. Introduction

Solid oxide fuel cells (SOFCs) possess unique advantages, including high efficiency (40%), minimal emissions, noise-free operation, and compatibility with a wide range of fuel sources. Consequently, they have been recognized as a highly efficient green energy technology [1,2,3]. Due to their high operating temperatures (500~800 °C) and solid oxygen ion conductor electrolyte, SOFCs can theoretically convert the chemical energy in almost all hydrocarbon fuels into electrical energy. Among these fuels, CH_4_ has been considered the most attractive fuel due to its abundant availability, narrow explosion limit range, low cost, and ease of storage [4]. In addition to natural gas reserves, biogas produced from anaerobic decomposition of organic material also represents a viable source with nearly equal concentrations of CH_4_ and CO_2_ [5]. The direct utilization of CH_4_ as a fuel offers several benefits such as reducing system complexity and costs, avoiding safety issues associated with fuel storage and transportation, and reducing greenhouse gas emissions. Therefore, it has become a hotspot in SOFC research [6].

Compared to conventional SOFCs employing yttrium-stabilized zirconia (YSZ) electrolytes, proton conductor SOFCs (H-SOFCs) have garnered significant attention from researchers in recent years. H-SOFCs operate at lower temperatures ranging from 500 to 700 °C, which enables quicker startup times without requiring expensive heat-resistant materials [7,8,9]. However, the direct utilization of CH_4_ fuel in H-SOFCs poses a major challenge since no oxygen ions can be transferred to the anode for direct oxidation of carbon-containing fuels. As a result, a large amount of unconverted CH_4_ remains, and carbon deposition is prone to occur on Ni-based anodes, resulting in rapid performance degradation.

In order to directly use CH_4_ in H-SOFCs, CH_4_ fuel was pre-reformed into CO and H_2_. Then, the syngas was fed into an SOFC anode, which not only improved the utilization of CH_4_ but also blocked direct contact between the CH_4_ and the anode to avoid carbon deposition [10,11]. At present, most studies focus on adding a layer of a reforming catalyst on the surface of the anode to realize in situ reforming of the fuel [12,13,14]. These catalysts have the function of converting hydrocarbons into gases such as H_2_ and CO. This concept was first proposed by Zhan and Barnett [15]. After Ru-CeO_2_ was added to the outside of a traditional anode, a peak power density of 300~600 mW cm^−2^ could be obtained for a single cell at 670~770 °C. Afterwards, Hua added a Ni_0.8_Co_0.2_–La_0.2_Ce_0.8_O_1.9_ (NiCo–LDC) catalyst to the outside of a BaZr_0.1_Ce_0.7_Y_0.1_Yb_0.1_O_3-δ_ (BZCYYb) anode, and the cell operated stably at 700 °C for 100 h [16]. Adding Ni-based catalytic electrode particles could hinder gas diffusion and electron transfer, leading to an increase in impedance and affecting long-term efficiency. Han’s team found that the addition of Ni-Ce_0.9_Gd_0.1_O_2-δ_ (GDC) catalytic materials resulted in a higher interfacial resistance between the anode and the catalytic material, and the low porosity of the catalytic material hindered the diffusion of fuel gas, leading to significant concentration polarization in the cells [17]. Barnett also found that adding catalytic electrodes increased the limitation of gas transport, and the insufficient conductivity of the oxide electrode materials increased the interface resistance [15,18].

At present, most of the catalytic materials for on-cell reforming are supported Ni-based catalysts, especially nanoparticles, which have problems such as uneven distributions of active components and weak bonding with their supports and are prone to carbon deposition and deactivation at high temperatures [19,20]. Although several catalysts such as Ni/SiO_2_ [21] and Ni/Al_2_O_3_ [22] showed better Ni dispersion and smaller particle sizes via modified preparation strategies, the complicated operation conditions of on-cell SOFC systems lead to higher requirements for the stability and electrical properties of the catalysts. Perovskite oxide is a kind of novel inorganic material with unique physical and chemical properties, such as thermal stability, high catalytic efficiency, and electrical conductivity. It is widely used in catalysis and shows excellent performance [23,24,25]. The A-site ions are mainly rare-earth metal ions (La, Ba, Ce, Pr, etc.) with large radii and alkaline earth metal ions that play a role in stabilizing the structure and do not participate in catalytic reactions. The B-site transition metal ions in the bulk can be precipitated from the crystal lattice into highly dispersed metal particles separated on the surface of the perovskite substrate. This method results in a stronger metal support interaction and a more satisfactory anti-sintering ability than the impregnation method, which is also called in situ exsolution technology. In addition, A-site-deficient perovskites (A/B < 1) can promote B-site metallic element exsolution [26,27], as A-site deficiency provides a driving force for the reduction of doped metals such as La_0.52_Sr_0.28_Ni_0.06_Ti_0.94_O_3_ [27] and La_0.9_Mn_0.8_Ni_0.2_O_3_ [28]. However, the porosity and specific surface areas of perovskite oxides prepared by liquid-phase methods like the sol–gel process or solid-phase reactions are not sufficient due to high-temperature calcination.

In the present study, a La_0.85_Ce_0.05_Mn_0.9_Ni_0.1_O_3_ (LCMN) perovskite nanofiber with high porosity was synthesized with a modified electrostatic spinning method. It was imposed on a conventional Ni–BZCYYb anode for direct use of CH_4_ in SOFCs via on-cell CH_4_ reforming so that the catalytic electrode gas transfer obstacles that restrict SOFC performance could be overcome. And the electrochemical performance and carbon deposition of the formed LCMN/Ni–BZCYYb anode were investigated to evaluate the feasibility of on-cell reforming. For comparison, a La_0.85_Ce_0.05_Mn_0.9_Ni_0.1_O_3_ (LCMN) perovskite powder was prepared by the sol–gel method mentioned previously, and the catalytic materials for on-cell reforming were deposited on the outside of a Ni–BZCYYb anode.

## 2. Results

### 2.1. Material Characteristics

**Figure 1:**
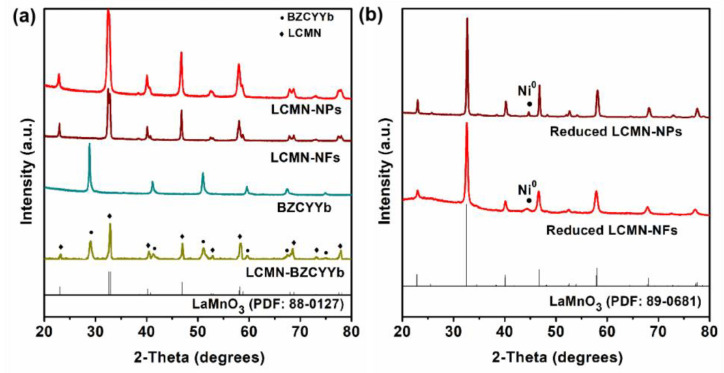
(**a**). XRD patterns of as−prepared materials, including LCMN−NPs (red line), LCMN−NFs (wine line), BZCYYb (dark cyan line), and mixed LCMN−BZCYYb (dark yellow line); (**b**). XRD patterns of reduced LCMN−NPs (wine line) and LCMN−NFs (red line).

Figure 1 illustrates the XRD results obtained from various powder samples. As-prepared LCMN-NPs and LCMN-NFs exhibited perovskite structures similar to that of LaMnO_3_ (PDF card: 88-0127) [29] without any additional phases present. To assess compatibility, BZCYYb was mixed with LCMN at a weight ratio of 1:1 and heat-treated at 1000 °C in air for 3 h. Figure 1a shows two typical sets of characteristic diffraction patterns in this mixed sample without any formation of extra phases, indicating chemical compatibility between the LCMN catalyst and the BZCYYb electrode material. After reduction in a pure H_2_ atmosphere at 600 °C for 3 h, with more and more oxygen loss and vacancy formation, the LCMN perovskite structure changed into an orthorhombic structure in the Pnma space group (PDF card: 89-0681) [30]. As shown in Figure 1b, the peak signal of the LCMN-NFs shifted to a lower degree after reduction, demonstrating obvious structural expansion, namely tensile strain [31]. In fact, when oxygen vacancies were introduced, the Coulombic repulsion between the neighboring B-site cations caused the crystal lattice to expand [32]. The LCMN-NFs, which displayed a structure and surface area that were more porous, may have been reduced to a higher extent with more oxygen vacancies. On the other hand, both the LCMN-NFs and LCMN-NPs primarily consisted of perovskite-phase LCMN, along with minor metallic Ni phase formation, suggesting successful exsolution of metallic Ni during reduction.

**Figure 2:**
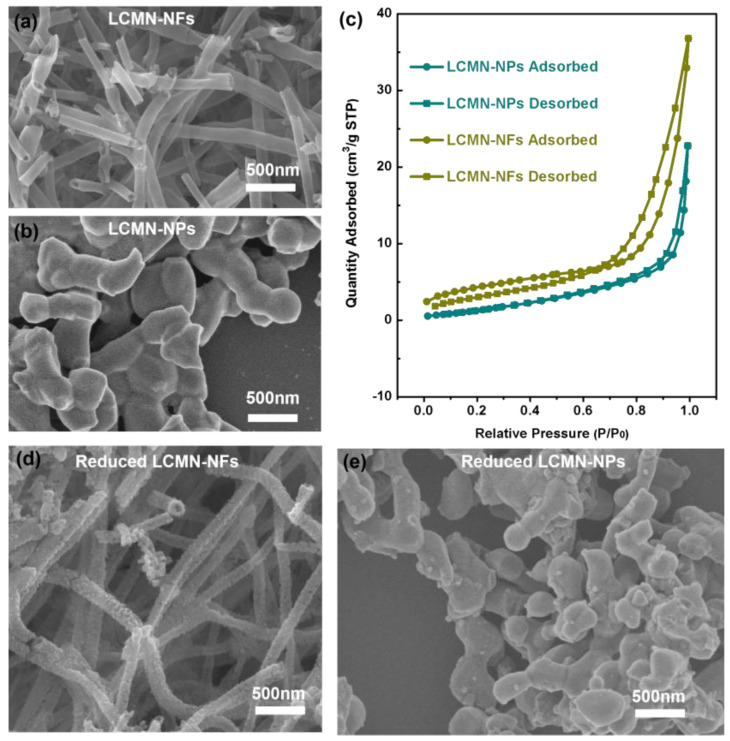
SEM images of as−prepared LCMN−NFs (**a**) and LCMN−NPs (**b**), N_2_ adsorption−desorption isotherms of LCMN−NFs and LCMN−NPs (**c**), and SEM images of reduced LCMN−NFs (**d**) and LCMN−NPs (**e**).

Based on the SEM images shown in Figure 2, the LCMN-NFs displayed typical porous nanotubes and the LCMN-NPs had a grainy structure with a smooth surface. As shown in Figures S1 and S2, the elemental mapping data from the EDS were gathered, and this further verified the uniform distribution of the elements La, Ce, Mn, and Ni in both the LCMN-NFs and LCMN-NPs. At the same time, the specific surface area of the LCMN NFs was 28.4 m^2^ g^−1^, which was larger than that of the LCMN NPs (11.2 m^2^ g^−1^) (Figure 2c). Apparently, after reduction, the microstructures of the LCMN-NFs remained almost the same, except for numerous metallic Ni nanoparticles that were uniformly distributed on the surfaces of the perovskite nanofibers (Figure 2d). Fine nanoparticles were also exsolved on the surfaces of the LCMN NPs. Based on the XRD and SEM characterization, the metallic Ni nanoparticles were demonstrated to be successfully exsolved onto the surfaces.

### 2.2. Cell Microstructures

**Figure 3:**
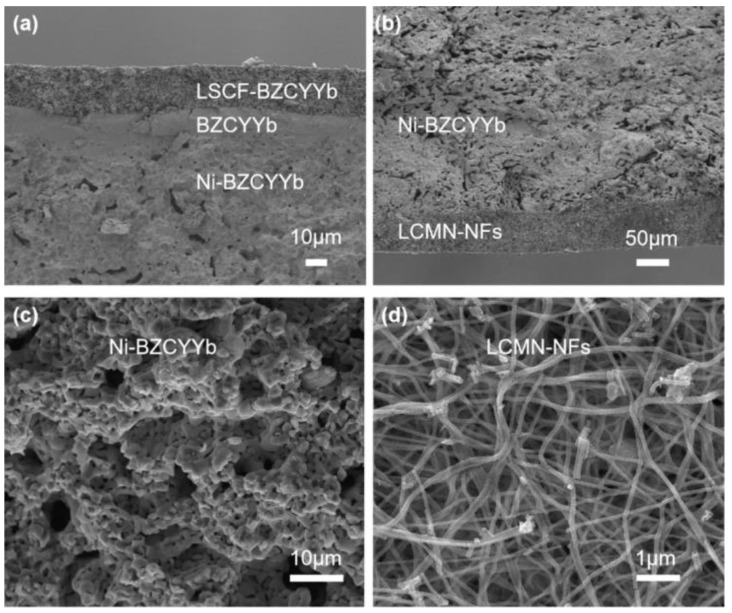
Microstructure of single cell: (**a**) LSCF−BZCYYb cathode/BZCYYb electrolyte/Ni−BZCYYb anode; (**b**) LCMN−NF catalyst layer/Ni−BZCYYb anode; (**c**) Ni−BZCYYb anode; and (**d**) LCMN−NF catalyst layer after reduction.

The cross-sectional microstructure of a whole cell reduced in H_2_ at 600 °C for 3 h is shown in Figure 3. The interfaces between the LSCF-BZCYYb cathode, dense BZCYYb electrolyte, porous Ni-BZCYYb anode, and porous LCMN-NF catalyst were well sintered. After reduction, the NiO in the anode was transformed into metallic Ni on a sub-micro scale, while a portion of the exsolved Ni in the LCMN-NFs formed uniformly distributed nanoparticles, as observed in Figure 2. Additionally, Figure S3 demonstrates that the porous LCMN-NP catalyst was tightly attached to the Ni-BZCYYb anode.

### 2.3. Cell Performances

**Figure 4:**
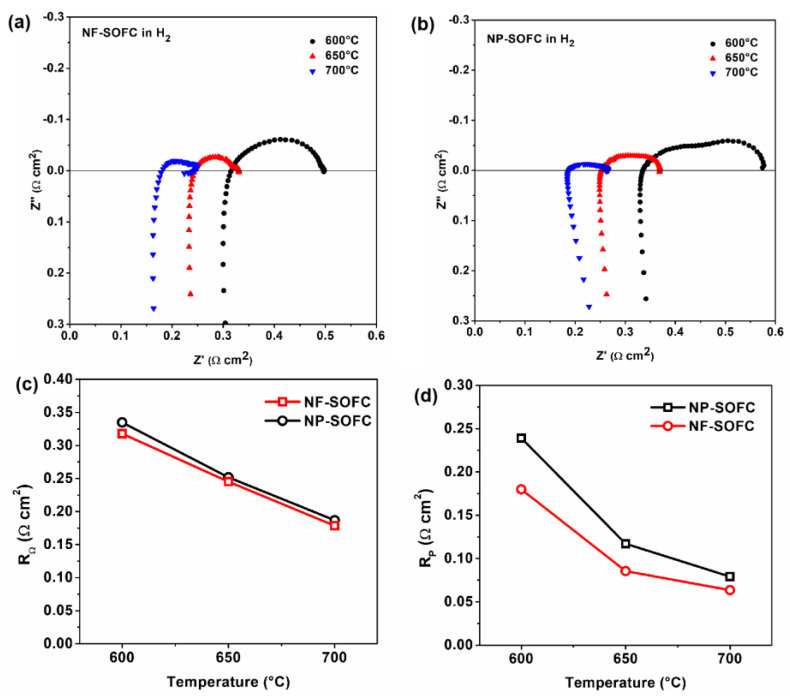
Open-circuit impedance spectra of NF−SOFC (**a**) and NP−SOFC (**b**) with H_2_ fuel at temperatures between 600 and 700 °C. Comparisons of ohmic (**c**) and polarization (**d**) resistances between NF−SOFC and NP−SOFC.

First, the electrochemical performances of both the NF-SOFC and NP-SOFC were evaluated using a mixture of 97% H_2_ and 3% H_2_O as fuel and air as an oxidant. The open-circuit EIS measurements of these cells at temperatures ranging from 600 °C to 700 °C are presented in Figure 4. Correspondingly, the NF-SOFC exhibited a similar ohmic resistance (R_Ω_) to the NP-SOFC but displayed a slightly lower polarization resistance (R_P_) (Figure 4a vs. Figure 4b). Considering that the two cells were assembled with the same electrolyte, it can be said that the addition of a layer of catalyst on the Ni-BZCYYb anode adversely affected the intrinsic cell performance. And the subtle difference in polarization resistance related to the electrochemical performance between the two cells fueled by 3% H_2_O–97% H_2_ can be attributed to the presence of porous nanofibers, which reduced additional resistance related to fuel gas transport within the anode region.

**Figure 5:**
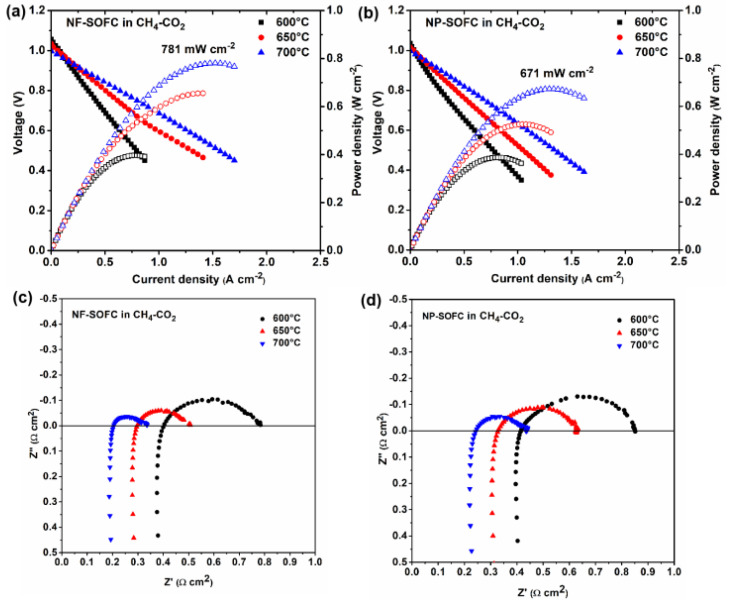
Electrochemical performances of NF−SOFC and NP−SOFC fueled by CH_4_−CO_2_: (**a**) I−V−P curves of NF−SOFC; (**b**) I−V−P curves of NP-SOFC; (**c**) EIS of NF−SOFC; and (**d**) EIS of NP−SOFC. (The solid symbols in (**a**,**b**) represent for Voltage and the hollow symbols represent for Power density).

When replacing the 3% H_2_O–97% H_2_ fuel with 50% CO_2_–50% CH_4_, the polarization resistances related to the electrochemical reaction for both cells increased because of the higher activation energy barrier of electrochemical CH_4_ oxidation compared to H_2_ oxidation. At the same time, it is not difficult to notice that the NF-SOFC outperformed the NP-SOFC (Figure 5). The maximum power density (MPD) of the NF-SOFC was observed to be 397, 655, and 781 mW cm^−2^ (Figure 5a), which was somewhat higher than that of the NP-SOFC at 386, 524, and 671 mW cm^−2^ (Figure 5b). Furthermore, the R_P_ of the NF-SOFC (0.132 W cm^2^) was lower than that of the NF-SOFC (0.132 W cm^2^) at 700 °C (Figure 5c,d). In addition, the OCV values of both cells remained close to each other within a range between 1.06 V and 0.99 V from 600 °C to 700 °C and decreased with increasing testing temperatures, which complied with thermodynamic laws and suggested good attachment between the cells and the cell holder.

**Figure 6:**
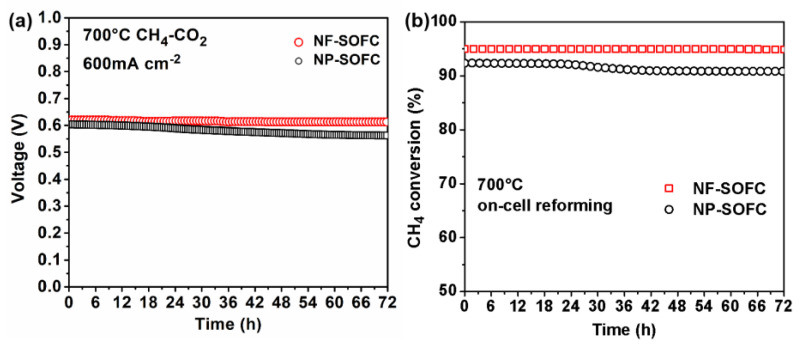
Performance durability of NF−SOFC and NP−SOFC fueled by CH_4_−CO_2_ at 700 °C for 72 h: (**a**) time-dependent cell voltage at 600 mA cm^−2^ and (**b**) time dependence of CH_4_ conversion.

To assess the stability of the cells, long-term discharge tests were conducted on the NF-SOFC and the NP-SOFC for 72 h at a current density of 600 mA cm^−2^ and a temperature of 700 °C (Figure 6). Similar to the maximum power density results, the NF-SOFC also demonstrated better stability than the NP-SOFC. In a 50% CO_2_–50% CH_4_ atmosphere, the NF-SOFC demonstrated remarkable stability, maintaining a voltage range of 0.61–0.62 V consistently throughout the entire test period. Conversely, the voltage of the NP-SOFC decreased slightly from an initial value of 0.60 V to 0.55 V after 72 h, exhibiting a decay rate of approximately 0.7 mV h^−1^, which highlighted a difference in performance stability between the two cells. The exhaust gas composition changes also reflected the stability performance of the NF-SOFC, which was monitored from the anode side during cell testing (Figures S4 and S5). Furthermore, CH_4_ conversion was determined based on these measurements (Figure 6b). CH_4_ conversion in the NF-SOFC reached 95.1% at 700 °C, which was higher than that achieved in the NP-SOFC (90.5%). This result suggests that LCMN-NFs with significantly increased surface area and active sites emerged as superior catalysts for the dry reforming process. This pre-reforming converted the majority of the CO_2_ and CH_4_ into H_2_ prior to reaching the Ni-BZCYYb anodes, where the electrochemical oxidation of H_2_ proceeded with a lower activation energy barrier compared to the direct oxidation of CH_4_. Consequently, the on-cell reforming processes on the NF-SOFCs reduced the impedance in the SOFCs and increased the power density.

**Figure 7:**
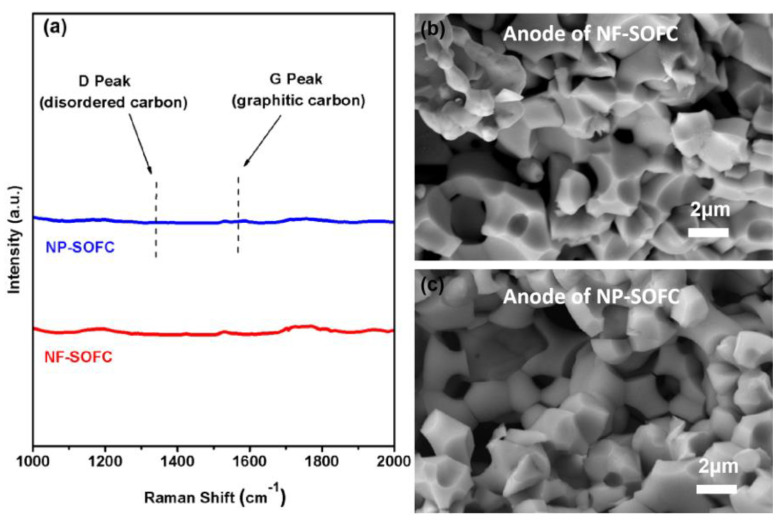
Raman spectra of anodes after stability test (**a**) and anode microstructures of NF−SOFC (**b**) and NP−SOFC (**c**) after long-term testing.

Raman spectroscopy was employed to detect carbon deposition in the Ni-BZCYYb anode of the cell following long-term testing, as Ni serves as an exceptional catalyst for cracking CH_4_, leading to carbon deposition. It is noteworthy that no discernible carbon peaks were observed at 1350 cm^−1^ and 1580 cm^−1^ (Figure 7a) for the NF-SOFC and NP-SOFC, respectively, which corresponded to the characteristic amorphous carbon D peak and the graphite carbon G peak, respectively. Subsequently, SEM was utilized to characterize the microstructure of the Ni-BZCYYb anode in both the NF-SOFC and NP-SOFC after prolonged testing. The anodes of both cells consisted of darker metallic Ni regions and brighter BZCYYb phases. The surface of the NF-SOFC anode appeared clean without any evidence of carbon deposition (Figure 7b). This absence further supports the potential of LCMN as a catalyst capable of almost completely converting CH_4_ and CO_2_ fuel into H_2_ in the on-cell reforming system. The formed H_2_ is oxidized electrochemically within the Ni-BZCYYb anode with an activation energy barrier lower than that of the direct oxidation of CH_4_. More CH_4_ conversion means more electrochemical H_2_ oxidation, enabling stable power generation and sustained performance stability.

## 3. Materials and Methods

### 3.1. Material Preparation

Using a typical electrostatic spinning method, La(NO_3_)_3_·6H_2_O, Ce(NO_3_)_3_·6H_2_O, Mn(NO_3_)_2_·4H_2_O, and Ni(CH_3_COO)_2_·4H_2_O with a La/Ce/Mn/Ni molar ratio of 0.85:0.05:0.9:0.1, together with polyvinylpyrrolidone (PVP), were totally dissolved into N, N-dimethylformamide (DMF), and the resulting solution was fed into a spinning instrument with a high voltage of 20 kV. Electrospun nanofibers were collected and subsequently calcined at 700 °C for 3 h in air to obtain the target LCMN nanofiber (NF) catalyst. Using a modified sol–gel method, La(NO_3_)_3_·6H_2_O, Ce(NO_3_)_3_·6H_2_O, Mn(NO_3_)_2_·4H_2_O, and Ni(CH_3_COO)_2_·4H_2_O were totally dissolved in deionized water. Citric acid was added as a complexing agent, followed by calcination at 900 °C for 3 h to form LCMN nanoparticles (NPs). To understand the Ni exsolution in the LCMN in a reducing atmosphere, the prepared LCMN NFs and LCMN NPs were reduced in H_2_ at 600 °C for 3 h.

The sol–gel method was also adopted for the synthesis of a La_0.6_Sr_0.4_Co_0.2_Fe_0.8_O_3-δ_ (LSCF) powder for the cathode and a BaZr_0.1_Ce_0.7_Y_0.1_Yb_0.1_O_3_ (BZCYYb) powder for the electrolyte as described previously. For the LSCF, calcination was conducted at 900 °C in air for 3 h, while for the BZCYYb calcination was conducted at 1100 °C in air for 3 h. All prepared powders were ball-milled before use.

### 3.2. Cell Fabrication

Anode-supported cells were fabricated using a dry press molding–screen printing–sintering process. NiO, BZCYYb powder, and corn starch were weighed according to the mass ratio of 55:45:15 and dissolved in anhydrous ethanol. After ball milling, the slurry was dried, ground, die-pressed at 20 MPa, and pre-sintered at 1050 °C in air for 2 h. A BZCYYb electrolyte paste with ethyl cellulose and a terpineol binder was screen-printed on one side of the dried anode before co-sintering at 1500 °C in air for 6 h. A cathode paste consisting of LSCF and BZCYYb at a weight ratio of 70 to 30 was then screen-printed on the surface of the sintered BZCYYb electrolyte, followed by sintering at 950 °C in air for 2 h to complete the fabrication of the anode-supported cell. Finally, an LCMN-NF paste was printed on the NiO-BZCYYb anode, followed by calcination at 1000 °C in air for 2 h. LCMN-NPs were also printed for comparison purposes. The cell had a diameter of approximately 13 mm, while the cathode had a diameter of approximately 8 mm. Furthermore, the cathode’s active area measured about 0.5 cm^2^, whereas the thickness of the anode was around 1 mm with an additional on-cell reforming layer measuring about 50 μm. Here, the cell with an LCMN-NF layer was defined as an NF-SOFC, and the cell with an LCMN-NP layer was defined as an NP-SOFC.

### 3.3. Material Characterization

Wide-angle X-ray diffraction (XRD) patterns were recorded on a Bruker D8 diffractometer (Bruker, Karlsruhe, Germany) using Cu Ka radiation (λ = 1.5406 Å) with a scanning angle range of 20°–80° at a scanning speed of 5° min^−1^ to examine the phases and structures in as-prepared LCMN, the chemical compatibility between BZCYYb and LCMN, and the chemical stability of the LCMN powder in a H_2_ atmosphere. The porous structures of the catalysts were characterized by a N_2_ adsorption/desorption (BET) method using an ASAP 2460 microporous analyzer (Micromeritics, Shanghai, China). Prior to characterization, the samples were degassed under vacuum at 200 °C for 6 h. Scanning electron microscope (SEM) images were obtained on a Gemini 300 field-emission scanning electron microscope (ZEISS, Oberkochen, Gemany). In addition, the microstructures of the cells before and after the electrochemical tests were also examined by SEM. Raman spectra were also investigated via a LabRAM HR800 Raman microscope (Horiba Jobin Yvon, Paris, France) to detect carbon formation in the anode.

### 3.4. Electrochemical and Catalytic Testing

The four-electrode method was employed for single-cell performance tests, including the measurement of current density (I)–voltage (V)–powder density (P) curves and electrochemical impedance spectra (EIS), which were evaluated using an electrochemical station. A Pt slurry was applied to the cathode surface as a current collector, while the assembled Ni foam was used for anode current collection. Prior to testing, the cell was attached to an Al_2_O_3_ tube (cell holder) using a ceramic sealing material and pre-reduced in situ by passing H_2_ through the anode at 600 °C for 3 h to reduce the LCMN layer and the NiO in the NiO-BZCYYb anode. In a typical test scenario, wet H_2_ (with 3% H_2_O and a flow rate of 50 mL min^−1^) served as a benchmark fuel, while a wet CO_2_-CH_4_ mixture (at a ratio of 1:1 with 3% H_2_O and a flow rate of 50 mL min^−1^) was used to demonstrate on-cell reforming effectiveness at temperatures ranging from 600 to 700 °C. The exhaust gas from the anode was analyzed using a gas chromatograph equipped with a molecular sieve column and a TCD detector during long-term testing. The conversion of CH_4_ was calculated by equation (1) based on the concentrations in the inlet ([CH_4_]in) and exhaust ([CH_4_]out) gases. After testing, both the morphology of the anode and the carbon deposition resistance of the entire cell were also observed.

(1) $$X_{{\mathrm{CH}}_{4}}=\frac{{{[\mathrm{CH}}_{4}]}_{\mathrm{in}}-{{[\mathrm{CH}}_{4}]}_{\mathrm{out}}}{{{[\mathrm{CH}}_{4}]}_{\mathrm{in}}}\text{×}100\%$$

## 4. Conclusions

In this study, we present the concept of on-cell CH_4_ reforming via SOFC power generation, in which CH_4_ is pre-converted into H_2_ using a reforming layer and electrochemically oxidized on a Ni-BZCYYb anode. In order to improve the morphology, porosity, specific surface area, and on-cell reforming performance of the reforming layer, we synthesized a LaMnO_3_-type perovskite LCMN nanofiber with a specific surface area of 28.4 m^2^ g^−1^, which acted as a reforming catalyst, via an optimized electrostatic spinning method. The optimization of the reforming layer led to significant improvements in fuel gas transport and charge transport within the anode. Thus, lower ohmic and polarization resistances were obtained for an NF-SOFC for both H_2_ fuel and CH_4_-CO_2_ fuel. These were key factors affecting the overall performance of the cell. When fed CH_4_-CO_2_ at 700 °C, the NF-SOFC displayed a maximum power density (MPD) of 781 mW cm^−2^, which was higher than that of the NP-SOFC (671 mW cm^−2^), and a stable discharge voltage of around 0.62 V was observed for 72 h. No carbon deposition was detected in the Ni-BZCYYb anode via Raman or SEM examination. This on-cell reforming system with a modified nanofiber as a reforming catalyst demonstrated stability during highly efficient power generation with hydrocarbon fuels.

## Data Availability

All data included in this study are available upon request by contacting the corresponding author.

## Supplementary Materials

The following supporting information can be downloaded at: https://www.mdpi.com/article/10.3390/molecules29153654/s1, SEM-EDX elemental mappings for LCMN nanofibers (Figure S1); SEM-EDX elemental mappings for LCMN nanoparticles (Figure S2); Cross-sectional microstructure of NP-SOFC (Figure S3); Time-dependent compositions of anode exhaust gas for NF-SOFC (Figure S4); NP-SOFC (Figure S5).
