# Supplementary material for: LaMnO3-Type Perovskite Nanofibers as Effective Catalysts for On-Cell CH4 Reforming via Solid Oxide Fuel Cells
Source: Molecules. 2024 Aug 1;29(15):3654. doi: 10.3390/molecules29153654 (PMC11313725; doi:10.3390/molecules29153654)
Supplement: Supplementary file 1 [file molecules-29-03654-s001.zip › molecules-3134433-supplementary.pdf]

Supporting Information

$LaMnO_3$-Type Perovskite Nanofibers as Effective Catalysts for On-Cell CH_4 Reforming via Solid Oxide Fuel Cells

Yangbo Jia [1], Tong Wei [1,*], Zhufeng Shao [2], Yunpeng Song [3], Xue Huang [4,*], Beila Huang [4], Chen Cao [1] and Yufan Zhi [1]

1 School of Materials Science & Engineering, Zhejiang SCI-TECH University, Hangzhou 310018, China; 2021316101121@mails.zstu.edu.cn (Y.J.); 2022316101093@mails.zstu.edu.cn (C.C.); 2023316101096@mails.zstu.edu.cn (Y.Z.)

2 China Industrial Energy Conservation and Cleaner Production Association, Beijing 100034, China; shaozhufeng163@163.com

3 Industry Development Center of Zhejiang Province, Hangzhou 310006, China; 15397109810@189.cn

4 Zhejiang Institute of Industry and Information Technology, Hangzhou 310006, China; 15067154645@163.com

* Correspondence: weit@zstu.edu.cn (T.W.); huangxue@zju.edu.cn (X.H.)

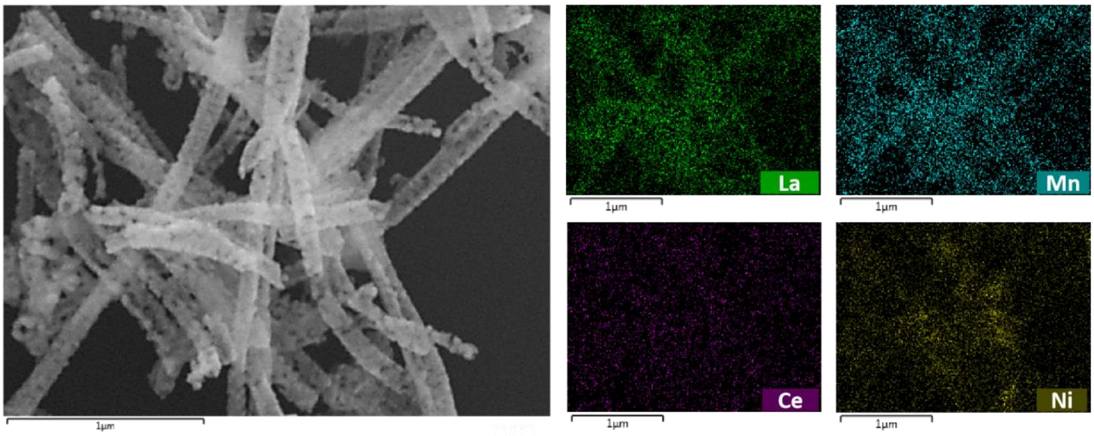

Figure S1. SEM-EDX elemental mappings for LCMN nanofibers.

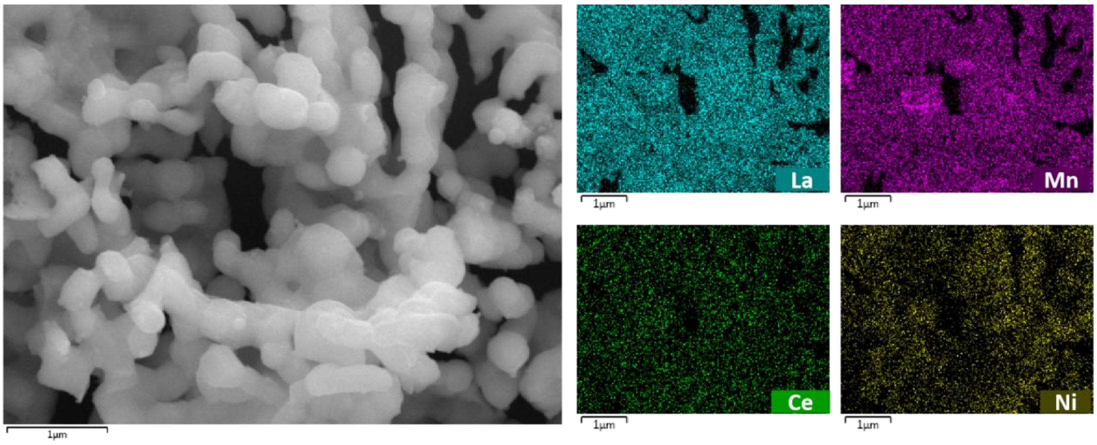

Figure S2. SEM-EDX elemental mappings for LCMN nanoparticles.

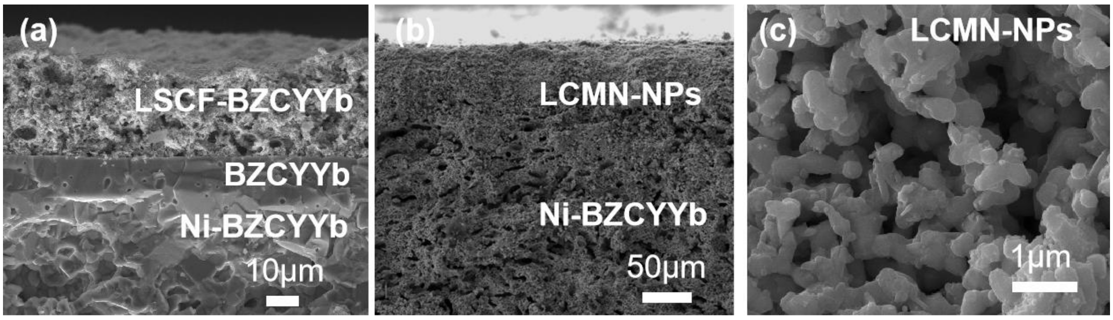

Figure S3. Cross-sectional microstructure of NP-SOFC: (a) triple-layer LSCF-BZCYYb cathode/BZCYYb electrolyte/Ni-BZCYYb anode; (b) double-layer LCMN-NFs catalyst layer/Ni-BZCYYb anode; (c) LCMN-NPs catalyst layer .

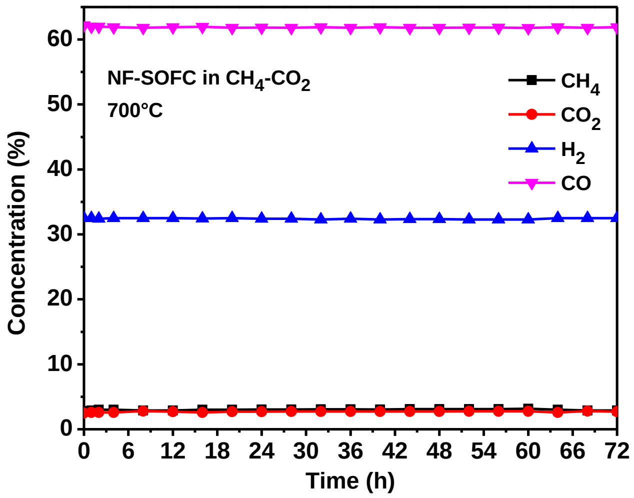

Figure S4. Time dependent compositions of anode exhaust gas for NF-SOFC fueled by CH_4-CO_2 at 700 °C and 600 mA cm^{-2} for 72 h.

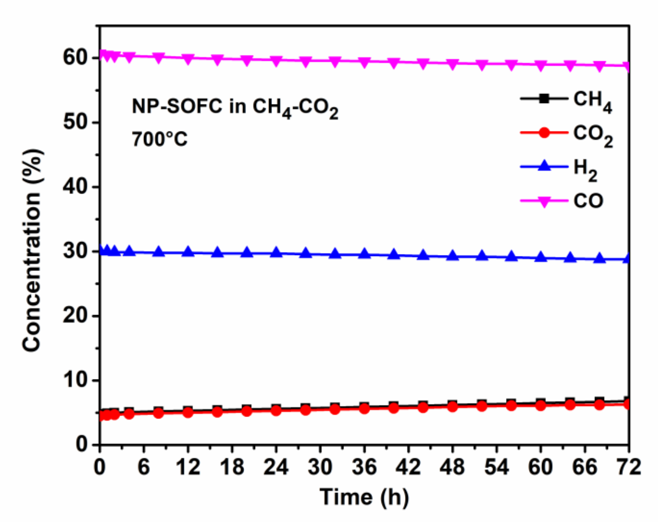

Figure S5. Time dependent compositions of anode exhaust gas for NP-SOFC fueled by CH_4-CO_2 at 700 °C and 600 mA cm^{-2} for 72 h.
